# Research Co-production Within Humanitarian Health: Reflections on Our Practice

**DOI:** 10.1177/19408447241241501

**Published:** 2024-03-24

**Authors:** Michelle Lokot, Thurayya Zreik

**Affiliations:** 1 4906 London School of Hygiene & Tropical Medicine, UK; 2 Independent Consultant, Lebanon

**Keywords:** co-production, research, humanitarian

## Abstract

Research co-production is recognized as important for humanitarian health actors, as part of the growing drive towards localizing and decolonizing aid. Despite recognition that co-production is challenging to implement, reflexive accounts of co-production efforts remain limited. In this paper, we critically examine the role of co-production within a multi-partner research collaboration in Lebanon involving multiple academic, civil society and government-affiliated partners based in the UK and in Lebanon. Through interactive reflection sessions and interviews with research team partners, we document how co-production principles were embedded into our project, explore contextual factors influencing the collaboration, identify successes and challenges to co-production and identify future opportunities for research co-production. We find mixed understandings of co-production between team members and siloed efforts to co-produce within our partnership. We identify key challenges to co-production including contextual factors related to Lebanon and COVID-19, institutional power dynamics, budgets, difficulties in engaging service users and availability of stakeholders to co-produce, while mapping examples of successful co-production in our project. Our study emphasizes the importance of ensuring shared understandings of the scope of co-production at the outset of projects and suggest the collaborative analysis processes provide a key opportunity for researchers to embed co-production principles.

## Introduction

This paper critically examines the role of co-production within a multi-partner research project seeking to support government and partners in health system strengthening for better mental health of Syrian refugees and host communities in Lebanon. The concept of co-production of research has become increasingly relevant for humanitarian health actors, amidst growing recognition of global power hierarchies which often result in “local” actors being excluded from decision-making within research collaborations, as funding continues to increasingly be skewed towards actors in the “North” (Sibai et al., 2019; Sukarieh & Tannock, 2019; Sweis, 2019). In humanitarian settings, the drive towards localizing and decolonizing aid has also contributed to greater mobilization around the concept of co-production. While there is greater awareness of the problems with traditional research collaborations, research co-production is also seen as complex and difficult to implement (Lokot & Wake, 2021a). This paper represents an effort to critically reflect on how co-production has occurred within a research partnership.

Co-production as a concept originated in the public sector to ensure less hierarchical partnerships for the delivery of goods and services (Ostrom, 1996). Co-production has varied definitions, which Smith and colleagues (2022) suggest is a reflection of the varied contexts and disciplines informing co-production. In their review of co-production definitions across different disciplines, Bandola-Gill and colleagues (2023) identify five key meanings of co-production that cover: the relationship between science and policy, mutual and collaborative knowledge generation that emphasizes local/indigenous knowledge, transdisciplinarity, bridging of knowledge across boundaries, and research use intervention. In their review of definitions of co-production in health and social care disciplines, Masterson et al. (2022) find that although definitions of co-production are constantly evolving, over 60 most commonly cited definitions list underpinning values of co-production to be “equity, power, and trust,” while the inclusion of service users in co-production may not always be present.

Bovaird and Loeffler (2013) emphasize the inclusion of service users in decision-making in their definition of co-production of public services as “professionals and citizens making better use of each other’s assets, resources and contributions to achieve better outcomes and/or improved efficiency.” McLean and colleagues (2023) define research co-production as an “umbrella term” to describe “an approach to generating knowledge where researchers work in partnership with research beneficiaries and/or research users” (2). They draw upon on Kothari and colleagues’ (2022) definition, which defines research co-production in health and health systems research as a “a model of collaborative research that explicitly responds to knowledge user needs in order to produce research findings that are useful, useable, and used” (1). References to co-production can be found in other forms of collaborative research, including integrated knowledge translation (IKT), an approach that is built upon the partnership of researchers who produce knowledge and those for whom the knowledge is meant to be of use (“knowledge users”) (Jull et al., 2017). Although also based on partnership, IKT and other forms of participatory research such as participatory action research differ from co-production in that the origin of co-production explicitly includes beneficiaries (such as service users, in the case of healthcare) in the production of public goods and service (Nguyen et al., 2020).

Smith and colleagues (2022) offer a typology of co-production, identifying three distinct types of co-production. The first is citizens’ contributions to public services, which focuses on how the public participate in the production/delivery of public services. The second (integrated knowledge translation) and third (equitable and experientially informed research) are similar in focusing on strengthening the process of research. However, Smith and colleagues (2022) urge that the use of one or more types depends on the situation, challenging the idea of a hierarchy and urging researchers to choose the most appropriate definition for the situation. In our study, we used the definition and seven key principles developed by Lokot and Wake (2021b) which is based on existing literature and focuses on co-production in research:

“Co-production in research refers to a horizontal partnership between researchers (both academic and non-academic) and active research participants to undertake research that can inform action. Co-produced research tackles unequal power dynamics, challenges existing knowledge production hierarchies, ensures more equal partnerships and shared decision making, emphasises reciprocity, promotes mutual capacity strengthening, ensures greater reflexivity and enables flexible ways of interacting and working across the research cycle.”

As a reaction to hierarchical processes of decision-making, co-production has been recognized as having potential to shift how knowledge production occurs (Durose et al., 2017; Rose & Kalathil, 2019), including the perspectives of diverse actors rather than just academics or researchers (Harries et al., 2020). Bell and Pahl (2018) suggest co-production has potential to destabilize and transform academia: “Against academics who claim to ‘know best’, this move to re-position knowledge within particular communities is an important task” (110).

Co-production is “profoundly relational” (Allen et al., 2019, p. 320), and requires intentional engagement with power hierarchies present within partnerships. Rose and Kalathil (2019) emphasize that a critical part of co-production is making visible existing power relations “as a first step to dismantling them” (38). Tackling power dynamics is often discussed in the literature as a particularly challenging aspect of co-production—and one which does not receive as much attention as it should within efforts to co-produce (Turnhout et al., 2020). Green and Johns (2019) note that enabling all partners to have equal power is less realistic in research contexts where hierarchies (such as that of Principal Investigator) might already be present, however, “sharing power” and creating spaces for people with the requisite expertise to be involved in decision-making is more achievable (61).

Co-production raises several key challenges within multi-partner collaborations. Flinders and colleagues (2016) describe co-production as “time-consuming, ethically complex, emotionally demanding, inherently unstable, vulnerable to external shocks, subject to competing demands and it challenges many disciplinary norms” (261), drawing attention to the multiple layers of challenges researchers must navigate when attempting to co-produce. Kimbell and Julier (2019) also discuss the challenge of institutional bureaucracies, asking: “How do researchers and participants deal with the dissonances produced when distinct bureaucratic regimes of accountability sit behind a variety of participants in co-production?” (6). Their question suggests that individuals simply having the will to co-produce may be insufficient because of the bureaucratic regimes individuals are accountable to. Within research, this raises the question of who holds funding for research, as this also inevitably shapes interactions and power, with donors having increasing influence over research scope (Fast, 2019). Power itself is challenged within research co-production because co-production “is a political process” (Kagan, 2013, p. 13), but it is not so clear if and how power dynamics can truly be unravelled through co-production (Farr, 2018). As Oliver and colleagues (2019) found, efforts to co-produce may often be confined to individuals at similar levels rather than covering all levels. Co-production is also fundamentally challenged by the research context itself: “what we set out to do can often be constrained by the realities of the challenges involved in undertaking the research” (INVOLVE, 2019, p. 4).

The popularization of the concept of co-production has led to misuse and misappropriation of the term (Lokot & Wake, 2021a), as well as tokenistic efforts to integrate marginalized groups such as service users (Crompton, 2019). Indeed, efforts to draw on “local knowledge” may brush over the power held by dominant groups, gatekeepers and elites, such that knowledge production may be less diverse than implied (Flinders et al., 2016, p. 271). Beebeejaun and colleagues (Beebeejaun et al., 2014) argue: “Simply applying the label of ‘co-production’ to research does not mean scholars are truly delivering on their desired aims” (12). However, it remains challenging, if not impossible, to evaluate if and to what extent research has been co-produced. The INVOLVE group, whose work on co-production has involved developing guidance and reflection questions to support institutions in reflecting on co-promotion, observe: “There is no gold standard of what co-produced research should look like and, at this stage at least, no criteria for assessing co-production” (INVOLVE, 2019, p. 4). The lack of clear understanding of what constitutes co-production—and what does not—means that some of the literature involves debates about what is real or true co-production (Farr, 2018). The notion of co-production occurring on a spectrum (Carter et al., 2019) has helped to soften the tensions about what counts as co-production, but this still remains an area of ambiguity.

While literature on research co-production continues to grow, there are limited examples of research co-production in humanitarian settings, as we found in previous research (Lokot & Wake, 2021a). The burgeoning literature emphasizes the value of co-production, as well as the real challenges and dilemmas of co-producing research in humanitarian settings. However, reflexive accounts of research co-production itself are limited within existing literature. Kirkegaard and Andersen (2018) suggest that the everyday interactions related to co-production need to be documented: “Fine-grained studies of real interactions are necessary if we are to move beyond the intentions and discourse of co-production towards a nuanced understanding of how it emerges as lived reality” (829). As such, this paper is a means of reflecting on the co-production process within a multi-partner research collaboration. It aims to (1) explore how co-production principles have been embedded into GOAL across all phases of the research from the perspective of different research partners; (2) explore the role of contextual factors in Lebanon (including COVID-19) that may have influenced our research collaboration; (3) identify successes and challenges to embedding co-production principles within GOAL; and (4) identify further opportunities to embed co-production principles into GOAL and future research collaborations.

The GOAL (“Supporting government and partners in health system strengthening for better mental health of Syrian refugees and host communities in Lebanon”) project (London School of Hygiene & Tropical Medicine, 2020) aims to support the government and partners in strengthening health system responsiveness to mental health needs in the context of protracted displacement in Lebanon, including the mental health needs of Syrian refugees and the Lebanese host community. Initially a three-year research project, GOAL began in February 2020 and was granted an 18-month extension until June 2024. The GOAL project is funded by United Kingdom Research and Innovation (UKRI) and the team includes multiple academic, civil society, and government-affiliated partners based in the UK and in Lebanon: the National Mental Health Program of Lebanon (Ministry of Public Health) (NMHP), the London School of Hygiene and Tropical Medicine (LSHTM), ABAAD, St Joseph’s University of Beirut (USJ), War Child Holland (Lebanon), and Positive Negatives/Beyond Text. The project consists of key Work Packages, designed collaboratively, and focusing on themes related to governance, financing, and capacity-strengthening. The governance and financing work packages involved qualitative and quantitative data collection by a research team, as well as data analysis and write-up with constant feedback processes among partners. Co-production was discussed as an important aspect of GOAL particularly during the project kick-off, that would require each partner to play a role in co-production at various stages of the project, with some more involved than others. Partners attended a training session on co-production principles and a small group of research team members developed a plan for embedding co-production into the research, with most effort focused on ensuring co-production during development of research protocols, design of data collection tools, coding, analysis, and writing up. As part of reflecting on co-production throughout the GOAL project, we discussed the different layers of power dynamics within our research collaboration, including those between academic, policy, NGO, and government actors, as well as dynamics between UK-based and Lebanon-based team members, and senior (more experienced) and junior (less experienced) team members. We discussed how, even within each partner organization, hierarchies related to experience, education, position, and seniority operate to further complicate power dynamics. Partners also recognized the challenges of co-producing research in a humanitarian setting like Lebanon, where apart from hosting large numbers of Syrian refugees over a decade, intersecting crises including economic pressures, political instability, the COVID-19 pandemic, and the 2020 Beirut blast have created a challenging context for humanitarian actors. Our reflections in this paper acknowledge these realities.

## Methods

Our study aimed to explore how different co-production principles were embedded during the first few years of the GOAL project, as part of our commitment to ensuring ongoing opportunities for reflection and learning on co-production during the project.

We used a collaborative process to generate data for this paper. We firstly facilitated a reflection session using an online collaboration tool called “Jamboard” in early 2022, which allowed members of the GOAL team to (anonymously) contribute their thoughts on power dynamics within the GOAL collaboration, what has worked well, the challenges of collaboration, and ideas to improve collaboration using sticky notes. This included reflection on the impact of COVID-19 and Lebanon’s economic collapse and volatile political and security situation on our research collaboration.

Following this initial reflection exercise, which helped to inform the development of topic guides, we conducted 14 interviews with members of the GOAL research team later in 2022. We identified participants from the different organizations, work packages, and roles/positions in GOAL to ensure broad representation. In interviews, using a semi-structured topic guide, we explored some of the themes from the reflection activity in more depth.

We completed inductive and deductive coding using “Dedoose” software during 2023 to identify the themes from the interviews. Dedoose was used as it supports synchronous analysis by people in different locations. Our coding was based on key issues within co-production literature as well as issues raised by GOAL team members during interviews. Based on this analysis, we developed a draft paper. We presented key findings from this draft paper to the GOAL team later in 2023, and invited anonymized reactions and feedback on the findings using “Jamboard,” again inviting the research team to anonymously post sticky notes in response to the findings. We incorporated these reflections into the draft paper which was shared with interview participants to invite further feedback and comments. In our findings, we chose to only indicate whether the participants quoted are Lebanon- or UK-based to preserve the anonymity of the participants.

This study received ethics approval from the London School of Hygiene and Tropical Medicine and Université Saint-Joseph de Beyrouth in 2021. Interview participants were asked to sign consent forms. A literature review was already conducted on co-production literature in 2020–2021 for the RECAP/GOAL co-production practice guide (Lokot & Wake, 2021b), which we draw on to write the paper.

Our reflections on the co-production process within GOAL are complicated by our own positionings within this research and our individual positionalities. As part of being reflexive about our own power and roles in this project, we recognize the challenges in disentangling our positionalities and identities from the research process (Orr & Bennett, 2009). ML, as an Australian of Sri Lankan ethnicity with experience as a humanitarian practitioner now works in a UK academic institution and benefits from the privilege associated with academia (Muhammad et al., 2015). As an early career researcher, she has some awareness of the power dynamics in academia that can affect research decision-making. Her role in the GOAL project has involved leadership of some components of the research. TZ is a Lebanese research consultant who had some experience in academia and in the public health field in Lebanon before joining the GOAL project. She joined GOAL in her capacity as a service user as well as researcher, and is aware of how her position as an “expert” service user is not necessarily representative of the majority of service users in Lebanon. She works with the Lebanon research team as a “lead” and understands the benefits of her previous academic experience as well as from having English language skills more generally.

## Findings

In this section, we reflect on how co-production has been realized in the GOAL project. We outline our findings across four broad themes discussed below: (1) understandings of co-production, (2) overview of co-production successes and challenges, (3) co-production across research stages, and (4) future opportunities for co-production.

### Understandings of Co-Production

Across interviews with GOAL team members, we found different understandings of co-production across different levels, including between partner organizations, between actors from different organizations, and between actors within the same organizations themselves. Most participants tended to refer to the involvement of multiple actors in decision-making within the research process when describing co-production, suggesting that co-production means “everyone has an equal voice” (Interview 6, UK-based) and that “no voice is above the others” (Interview 10, Lebanon-based). As part of emphasizing equitable decision-making, two participants specifically referred to the importance of involving communities themselves: “co-production would mean working with the community or the population in question” (Interview 11, Lebanon-based). Another participant reflected that co-production involves “a meaningful power shift to people who really have a deep kind of contextual understanding…” (Interview 9, UK-based). Participants also reflected on the blurred lines between co-production and decolonizing research.

Multiple participants also mentioned the idea of challenging traditional knowledge production through co-production: “co-production challenges like this basic idea that it’s not only that the academics - they know - but also people and their lived experiences is as rich” (Interview 8, Lebanon-based). A few participants explicitly referred to power, suggesting co-production involved “acknowledging that there are different power hierarchies between individuals, researchers, communities, stakeholders… [And] ensuring that these hierarchies are more flat” (Interview 4, UK-based). Co-production was also described as a “taking active steps to equalise the playing field” (Interview 1, Lebanon-based)—a way of levelling the research process.

Some participants, however, used the language of “collaboration” to describe co-production: “co-production is about getting more collaborative ways of working… a chance for everyone to be involved” (Interview 10, Lebanon-based). One participant in particular felt collaboration was a more helpful broader category to refer to, rather than specifically talking about co-production, suggesting, “co-production is one of the activities you could do as a collaborative aspect” (Interview 13, Lebanon-based).

A few participants focused on “outputs” when describing co-production rather than the process of research: “Co-production is when you work together with someone to produce an output, an outcome” (Interview 13, Lebanon-based). Another participant suggested co-production was more about all stakeholders having a unified goal: “It’s just aligning really the actors alone, [to] what would serve ultimately the goal of research” (Interview 12, Lebanon-based).

### Overview of Co-Production Successes and Challenges

Participants discussed a range of successes and challenges related to co-production, which we group across eight categories below: (a) how power is considered, (b) funding situation, (c) governance, (d) service user inclusion, (e) decision-making, (f) Lebanon context, (g) COVID and Zoom impacts, and (h) capacity-strengthening.

#### How Power is Considered

During interviews, participants discussed how they perceived power dynamics within GOAL. Multiple participants discussed how academia reflects particular power dynamics, which have shaped the direction of GOAL:

“Academic institutions are full of power hierarchies and expectations for what counts as success and what counts as a positive outcome and so that often drives some of the interventions and the focus on publications. So I think that definitely is a factor that has influenced GOAL and has shaped our focus on what is produced (Interview 6, UK-based).

Participants discussed how academia creates hierarchies based on academic experience, giving power to people who have PhD degrees. One participant described feeling part of the “inner circle” of the GOAL project, due to their academic status (Interview 4, UK-based). During the feedback session, one participant reflected on the importance of making visible power dynamics: “In order to know how to tackle power imbalances, it’s important to define what the power imbalances are that we are talking about.”

Participants also discussed other types of hierarchies within GOAL, for example, the power held by lead actors of each partner organisation and by workstream leads: “the people who are leading the protocols, it’s natural, because these are the people who are more powerful, and can speak on the updates to their project and can ask questions” (Interview 10, Lebanon-based). This participant referred to the “very strict hierarchies” each partner is operating within, suggesting that this “bleeds over into the project” and may still be difficult to unravel. Another participant reflected: “I think for a lot of people this is a new way of working and I think that it’s a different way of thinking that everybody is unfamiliar with” (Interview 5, UK-based). A few participants reflected on how power may shift depending on which partner organizations are involved, suggesting the power dynamics are most constant in interactions with partner staff who are directly involved in collecting data.

Multiple participants discussed how power hierarchies between different partners in GOAL have made it difficult to follow-up with more senior partners holding higher positions in leading organizations, resulting in “bottle-necks.” One participant described the challenge in following up with senior partners, reflecting: “we often didn’t feel like we could chase them, or there was some anxiety on asking them to respond… we felt nervous about harassing a senior person and asking them, ‘Why haven’t you responded?’” (Interview 6, UK-based). Another participant reflected that “in terms of the power differential, [they] didn’t feel comfortable chasing” (Interview 2, UK-based).

These reflections on power dynamics that are present in any research collaboration draw attention to the challenges of using co-production principles. A few participants also reflected on the fact that due to the efforts to reflect on power and challenge normal patterns of decision-making in GOAL “there’s a tendency to maybe understate or not be as critical of the power hierarchies. So to be, ‘Oh yes, everything is really equal. I feel like I’m really involved in the decisions’. When we are still within any research collaboration, there’s still that reality of, who holds the funding? Whose name is ultimately on the project, and who is ultimately responsible and accountable to the funder?” (Interview 6, UK-based). Other participants also reflected on how partners in GOAL may be “over appeasing” (Interview 1, Lebanon-based).

While the majority of participants reflected broadly on power dynamics within the GOAL project, a couple explicitly did not use power to frame dynamics between partners. One participant suggested that dynamics in GOAL were not about a power dynamic but was “more of a question of trying to understand each other” because each partner is “coming from very different perspectives with different priorities.” They felt this was more about “just the challenges of research” rather than power (Interview 3, UK-based). Another participant felt they didn’t feel power dynamics were present in GOAL, commenting “I don’t find it as an issue” and also reflecting they didn’t feel there was much discussion of power generally (Interview 13, Lebanon-based).

During feedback sessions, there were mixed perspectives on whether power has been explicitly discussed during the project, with some partners referencing attending reflection sessions on power and others saying that this could have been integrated more into activities. These different perspectives may be a reflection on where particular GOAL members were situated within the partnership. As one participant reflected, “these conversations have been happening among the lower ranks… and less among those who actually have power.” They commented that more senior partners “are still a little bit distanced from some other conversations about power,” however “need to be included in these conversations because they are often the ones who hold the power” (Interview 6, UK-based). During interviews, it tended to be GOAL members who were more involved in operationalizing the research who discussed the steps that were taken to challenge power dynamics. For example, participants involved in coding and analyzing data collaboratively mentioned this as an example of challenging normal power dynamics within research. They discussed how reflection sessions, small group discussions, and anonymous Jamboard activities helped them to reflect on power and positionality.

#### Funding Situation

Participants generally shared the view that the nature of the funding and the budget of the GOAL project allowed for greater flexibility within the research process itself, and that this was an enabler to co-production: “[T]he way that it’s structured particularly around finance and budgets means that the partners involved have a lot of say and choice in what they’re doing. So it’s less top down from us which is really nice” (Interview 5, UK-based). The same participant mentioned that partners receive a “really sizeable chunk of the budget” with flexible budget lines, allowing them greater flexibility to choose the activities they preferred to participate in and implement, which also helps to “at least lay the foundation of co-production, if not achieve it” (Interview 5, UK-based).

The UKRI, the funder of the GOAL project, was described as “extremely flexible.” This flexibility was said to have allowed for the integration of different ideas at various points in the research process, and to have made “a massive difference if we’re genuinely trying to do research, kind of, equitably and following principles of co-production, because it provides that flexibility by the funder it allows flexibility within the research and supports co-production” (Interview 7, UK-based).

Despite recognition of the general flexibility and equity in budget decision-making within GOAL, there was also recognition of LSHTM as lead actor. Participants noted that “if we’re discussing budget, certainly London School has a more prominent role” (Interview 13, Lebanon-based), and that there is an inevitable power differential that stems from being tied to British funding, but that with regards to GOAL, “I’m not sure much could have been done about that” (Interview 3, UK-based). Decision-making ability related to some funding decisions was described as limited to higher-level management, including decisions related to contracts. For example, co-production was also found to be difficult to implement during writing contracts, which usually follow a “top-down structure” (interview 5, UK-based). Despite this, the terms of reference for engagement in GOAL were described as having been co-produced between partners but not necessarily between staff within partner organizations.

Participants shared that following the UK ODA (Official Development Assistance) cuts to overseas aid funding, which resulted in severe cuts to the GOAL project budget, discussions were “constructive” between all partners despite challenges (Interview 11, Lebanon-based). Participants described how partners’ voices were taken into consideration: “I think it’s been very transparent and I think that everybody has had an opportunity to… it gave us an opportunity to rethink the budget completely and I think everyone was involved in that decision and continues to be involved in that decision in a way that can sometimes be difficult to manage” (Interview 5, UK-based). A participant mentioned that efforts were made to include all partners when redesigning the project after the budget cuts.

#### Governance

Participants described efforts to integrate co-production on the governance level. One participant gave the example of having “different people responsible for governance of the project, so not only academics, but also people from the Ministry of Health” (Interview 4, UK-based). Participants also shared the example of the GOAL Advisory Committee, which includes service users, and described this committee as a “big step” towards co-production and towards “hearing less well-heard voices in a project like this” (Interview 5, UK-based). Another participant added that co-production was built into the GOAL structure “through having great representation on the Advisory Committee” and inviting service user perspectives to comment on various aspects of the project, including dissemination (Interview 7, UK-based).

Participants also gave the example of the Management Group Meetings as facilitating co-production by facilitating more “inclusive” (Interview 11, Lebanon-based) decision-making. One participant reflected that although the PI “is always going to have ultimate accountability” that “I hear the partners’ voices a lot more than perhaps I have done in other projects” (Interview 5, UK-based). However, another participant mentioned that partners work “independently” apart from the Advisory Committee and Management Group meetings, with less interaction than at the beginning of the project before the COVID-19 pandemic.

#### Service User Inclusion

Although service user involvement was present during several stages of the project, participants noted a lack of inclusion of service user and refugee perspectives overall, and that “more work could have been done on that in terms of involvement in the project structure” (Interview 7, UK-based). Participants referred to “some obvious gaps” in the inclusion of representatives of the Syrian refugee community and mental health service users during the proposal writing and development stage (Interview 7, UK-based). Another commented that the project was “lacking any co-production” with Syrian refugees in particular (Interview 11, Lebanon-based).

Participants gave the example of service users being present at the project kick-off meeting as a step towards co-production. However, many participants noted that the service users who were involved were “experts” (Interview 1, Lebanon-based) and that there was a lack of diverse representation of service users who were “typical” service users (Interview 11, Lebanon-based), including service users from different socioeconomic backgrounds and different levels of literacy, as well as a lack of service users who are refugees. When describing the scoping and planning stages of the research project, one participant emphasized the lack of involvement of non-professional service users:

“[I]t’s easy to integrate a service user who has a master’s degree than someone who has a less education or isn’t going to be engaging with the participation... framework for example (…) I think it’s not a perfect process and we definitely could have done more at the beginning. I think maybe there was a bit of overreliance saying, ‘Oh yes, we have service users involved in the project’, but without really critically reflecting on, ‘Okay, but who are these service users?’ These are different to their regular service [users], it’s like an exceptional person with a very specific knowledge and interest in the topic (…) And so I think we rested on our laurels a little bit in terms of ticking the box…” (Interview 6, UK-based).

One participant pointed out that there may be other groups of service users who may have been excluded from participation in decision-making, such as people who have mental health needs but cannot access services, and actors within GOAL who may use services, but have not been “designated” as service users within the project (Interview 11, Lebanon-based).

Participants agreed that involving service users and refugees was generally challenging, and that the “capacity to do it was not easy” (Interview 12, Lebanon-based), especially when trying to do so while avoiding being “tokenistic” (Interview 7, UK-based). One participant described the difficulty of relying on the newly founded service user association in order to reach service users, suggesting that using different pathways of participation outside the service user association might have reached more service users. A participant mentioned that the GOAL advisory committee (which includes service users) is a way to attempt to address the gap in the inclusion of service users and refugees.

Although one of the National Mental Health Program’s stated aims within the GOAL project was to understand how to increase service user participation, some participants expressed the perception that the NMHP was representing service user perspectives rather than service users themselves being involved: “We had to rely on, kind of, [NMHP Leadership] perspective on that but it would have been really nice to have been able to talk to people from the communities or community leaders” (Interview 3, UK-based). One participant shared the belief that because the project focus is supporting mental health governance in Lebanon, the National Mental Health Programme was the “direct beneficiary” of the GOAL project rather than service users (Interview 12, Lebanon-based).

During the feedback session, one participant reflected that perhaps the intention of GOAL was just to co-produce between the different partners rather than also with service users—in which case we were more successful. Others however recognized the intention from the kick-off meeting to ensure service users were part of the research process. For the NMHP, for example, understanding how to better reflect the voices of service users was a motivating factor for conducting research on participation within the GOAL project. There was some suggestion as well that at the outset more effort should have been made to explicitly define who the service users were and to develop a specific service user group or advisory group only with service users to provide input throughout the research.

#### Decision-Making

Participants generally agreed that most decision-making in GOAL was collaborative and shared between partners. One participant described decision-making as “equitable” (Interview 13, Lebanon-based), while another described the decision-making process as “very collaborative (…) [and] has been done always in conjunction with the partners across institutions and countries,” continuing to say that the process was “fair and equitable and transparent and well-communicated” (Interview 3, UK-based). A participant mentioned that she “always feel [s] that there is room for flexible decision-making process” (Interview 10, Lebanon-based). Participants mentioned that the communication and engagement in GOAL resulted in a greater level of shared decision-making. For example, WhatsApp groups and email are used to make sure that people are “not just say included but they actually have a front seat in the decision making” (Interview 2, UK-based). Another participant mentioned that having more knowledge about the research process and structure of the project allows for greater involvement in decision-making.

However, some partners expressed the need for more clarity around the decision-making process, “so that everyone knows theoretically what the kind of roles and responsibilities are, and then, as usual, can challenge them and they can be changed” (Interview 9, UK-based).

Despite the fact that decision-making space is usually shared with all partners, “inevitably (…) the decision making ultimately is still being made often by the Leads of each of those partners” (Interview 7, UK-based). According to a participant, many actors still defer to the PI: “there’s still a culture of deferring upwards and a kind of before just making a decision or even as a group I think people feel comfortable or seem to feel comfortable checking and being sure that what they have proposed is okay” (Interview 5, UK-based). One participant mentioned that “bigger decisions,” such as decisions related to project management, were still made “very inclusively” (Interview 11, Lebanon-based). According to one participant, “there’s no partner that has more decision-making” (Interview 13, Lebanon-based). However, some partners (such as the LSHTM) were viewed to have greater decision-making power than others on topics like finance, although this was not necessarily perceived as negative.

Several participants gave the example of decision-making being shared and collaborative during the budget cuts: “[It] felt like that was a really fair and equitable decision-making process that was done respectively. So that’s probably when I felt most included. I didn’t necessarily know how that decision making would take place and I think I probably just thought that PIs would decide and tell us, but it felt like it was a bit more inclusive which was good” (Interview 6, UK-based).

Participants identified several challenges related to decision-making. For example, one participant mentioned that factors such as “the urgency of completing the proposal” might have led to some actors being excluded from the decision-making process (Interview 13, Lebanon-based). The decision-making process was also described to be sometimes “slow” or “very deliberative,” as well as time-consuming (Interview 3, UK-based). Another participant also attributed delayed decision-making to poor responsiveness of some actors who are overloaded: “Sometimes there’s three months of silence. It’s been quite stop and go for like a number of very understandable reasons all out of their control” (Interview 2, UK-based).

In some cases, participants shared the view that the time-consuming nature of the deliberative process meant that “sometimes executive decisions have to be made, and that’s okay.” (Interview 1, Lebanon-based):

“There’s a trade-off. It’s really good to be inclusive and deliberative, but then maybe one or two needs to balance that against efficient use of resources and maybe not everyone needs to be involved in that process. It can be delegated to a smaller group, so that it doesn’t become a very costly process in terms of time, and also the more people involved in decisions sometimes more complicated the process…” (Interview 3, UK-based).

During the feedback session, there was some discussion of the tension between authority and leadership that are needed in research more broadly, at risk of creating “anarchy” and “outright abuse of power.” Partners suggested leadership should be seen as a positive and something that is needed, and that using power can also occur without it being negative.

Despite co-production being intended to create a space for everyone to communicate openly and non-hierarchically, we identified that this is difficult to implement in practice because of team members being used to more hierarchical ways of working. For example, staff who are not usually given opportunities to share their views may not feel comfortable when suddenly invited to give their opinions. At times, these additional spaces for participation and leadership were unexpected, as one participant explained: “I was surprised a lot of times in the beginning, like, ‘Oh, you want me to present in this meeting?’ Like, really?” (Interview 1, Lebanon-based).

#### Lebanon Context

Participants described a high degree of flexibility in the GOAL project when accommodating for the challenges that arose from working within the Lebanon context. Many participants gave the example of the Beirut Blast, when a decision was taken to “hold off” on GOAL-related tasks for a month to allow time for the Lebanon team to cope with the crisis: “I think that was a good decision that reflected consideration of the needs of the partners rather than our deadlines or what the funder wants” (Interview 6, UK-based).

Participants also discussed flexibility in accommodating for challenges related to Internet access and electricity cuts in Lebanon: “[O]ur research teams are facing huge challenges and accessing electricity to have the meeting with us can cause a lot of stress. And so I think it has required us to rethink some of our ways of working, to change the plan sometimes, and be also more flexible” (Interview 6, UK-based). Another participant added, *“*I mean, to be honest I’ve been amazed at how responsive the team have been in the circumstances because it seems really difficult. I mean, we had like a call when I think [name] was joining from a café because he had no electricity in his apartment” (Interview 3, UK-based). However, according to one participant, Internet and electricity access challenges still had a negative impact on the co-production process:

“Actually, the internet and electricity was really stressful factor that it really affect me. Sometimes it was really hard to have internet all the time or good connection (…)and during COVID it was not eligible to go to offices. And our houses, in some areas in the north Lebanon, we didn’t have electricity all the time(…) So I tried different ways to have internet or to have connection (…) And this affected the meetings, and this affected the co-production process…” (Interview 14, Lebanon-based).

Participants discussed other ways in which the difficult Lebanon context and economic crisis affected the ability of Lebanon staff to participate in the project in general: “You know, food shortages, I mean, just seemed unbelievable and you could feel that mentally that was really putting a strain on people, but that they were really keen to continue with the research and have that focus which was quite inspiring really” (Interview 3, UK-based). One participant mentioned that the crisis affected the motivation of the Lebanon staff in general, which in turn led to them having less energy to participate or give their input. One participant discussed how managing challenges makes it difficult for the Lebanon team to participate and thus undermines their involvement in GOAL: “I think what kind of ended up happening is that it then becomes LSHTM driven because we’re not managing, having to manage the same challenges” (Interview 5, UK-based). Another participant agreed that the crisis affected participation in the project due to the need for Lebanon staff to focus on personal needs, and being “not really able to think of our professional needs and our contribution to the project, as much as we are worried about other things in life, like basic care, basic needs, and yes everything” (Interview 10, Lebanon-based). Another participant discussed the impact of the economic crisis on the availability of resources, which in turn “affects what people are able mentally and physically to do and it’s obviously a very, very difficult situation for the colleagues” (Interview 3, UK-based). Despite these challenges, one UK-based participant mentioned that they “don’t get a sense of how disruptive things are” because of the Lebanon team consistently delivering throughout these challenges (Interview 7, UK-based).

#### COVID and Zoom Impacts

The COVID-19 pandemic presented challenges to co-production within the GOAL project in general. Travel restrictions and remote working arrangements were found to have affected the ability to build personal relationships, which were seen as important for building rapport within the research team. One participant noted that despite the flexibility of the GOAL project to accommodate the challenges related to COVID-19, the shift to remote work denied actors of “important moments in the life of a group or a team” such as “site talks and the coffee time and lunch” (Interview 12, Lebanon-based). The effect of remote work on personal relationships, a key “ingredient” of co-production, was described to “restrict” co-production, by “[making] the relational aspects much more challenging to kind of grow and develop” (Interview 9, UK-based).

Participants mentioned how using Zoom impacted co-production. Using Zoom was described as producing unequal power dynamics, by affecting participation in speaking in meetings. Participants mentioned challenges in accessing Zoom that may be a barrier to participation, such as not having access to a phone or a quiet space to “sit in their house to be able to do a meeting or whatever it is*”* (Interview 2, UK-based). Another participant also mentioned that privacy could be a challenge when using Zoom: “I know that sometimes in our meetings and our ROs [research officers], they wouldn’t want to talk because I’m, you know, there’s so much like, you don’t have privacy when you have to be on Zoom” (Interview 1, Lebanon-based). In one case, Zoom increased awareness of power: “[S]ometimes you well actually, I’m kind of aware like I simply trying like jump on a call and chucking my voice. And I’m aware of, like what the kind of power dynamics of that. I’m aware of who’s speaking and how much they’re speaking…” (Interview 9, UK-based).

However, other participants mentioned that despite challenges, some aspects of remote work were found to have enhanced co-production practices. According to one participant, the relationships and collaboration online were “pretty effective” during the protocol development and planning periods, although they expressed that data analysis periods may need more face-to-face interaction (Interview 3, UK-based). Another participant also noted that “we were still able to connect even if we did not see each other face-to-face and we were able to get the work done” (Interview 8, Lebanon-based). One participant made the argument that the COVID-19 pandemic enabled the application of more co-production practices, such as flexibility: “[P]erversly I think the Covid pandemic has further allowed greater flexibility because it’s just harder for any funder to rigidly impose a certain original project plan” (Interview 7, UK-based). Another participant agreed that the use of Zoom “sometimes can flatten some of the power hierarchies” by allowing space for otherwise quieter actors to participate (Interview 2, UK-based).

The use of Zoom was also perceived to have had positive effects on participation: “it’s allowed perhaps more regular contact than would have happened prior to Covid in the sense that, you know, our use of Zoom or Skype would have been probably much less” (Interview 7, UK-based). Another participant added that without the use of Zoom, “we wouldn’t have had this much participation from the ROs, because they’re all based in different parts of Lebanon” (Interview 1, Lebanon-based). Other participants expanded on this and shared the belief that the use of Zoom allowed for greater participation of different actors by offering alternative channels for communication such as chat boxes:

“ [I]t’s a lot easier maybe to sometimes type a message. And so it did feel like some of those barriers to participation (…) also came down a little bit because of COVID and hopefully made it easier in some ways for us to collaborate, and to hear voices that we might not otherwise hear” (Interview 6, UK-based).

#### Capacity-Strengthening

Across interviews, participants indicated that capacity-strengthening efforts within GOAL have been helpful and appreciated, providing examples such as the gender workshop, qualitative methods workshops, and training content on the Lebanon MHPSS context. A few participants suggested capacity-strengthening could have been more “mutual” with greater involvement from the NMHP and other Lebanon partners such as USJ. LSHTM partners in particular recognized the need for more training on “contextual knowledge” for the LSHTM team (Interview 8, Lebanon-based). One participant however noted that the fact that LSHTM was “driving” capacity strengthening was because the Lebanon partners “just don’t have the time to put together a two-day training” compared to LSHTM staff who might have more space to develop content (Interview 6, UK-based). One participant indicated they could have benefited from a common capacity-strengthening plan to have a sense of all the trainings occurring across the workstreams (Interview 13, Lebanon-based). During feedback sessions, one participant reflected that capacity-strengthening could also be less formal (not just about trainings) and could have occurred more frequently from Lebanon-based staff to London-based staff.

### Co-Production Across Different Research Stages

In this section, we outline efforts to co-produce in GOAL across each research stage: (a) scoping, (b) design, (c) data collection, and (d) analysis and writing.

#### Scoping

Participants gave several examples of co-production in the scoping phase of the GOAL project, describing the process as inclusive of different stakeholders. One participant explained that because GOAL was co-designed with certain partners, and with the NMHP in particular, rather than solely by researchers in the UK, the project was “more relevant to their needs” (Interview 7, UK-based). Another participant stated that considering that the NMHP was a “beneficiary or focus of the research,” their inclusion in the scoping and planning stages was important for the co-production of the project (Interview 11, Lebanon-based).

When describing the study design, one participant mentioned that the process was inclusive; however, inclusion was less present during the proposal-writing stage: “what we haven’t done in the beginning was to shape the proposal together” (Interview 4, UK-based). Other participants noted that co-production was integrated into the project after the study had already been designed “a little bit later once some of the decisions had already been made,*”* instead of at the beginning of the process (Interview 6, UK-based). Another participant also mentioned that it has felt as though co-production had been “retro-fitted” into the project (Interview 5, UK-based).

Several participants gave the example of the initial “kick-off meeting” when discussing co-production during the scoping and planning stages. According to one participant, the inclusion of mental health service users in the meeting was a way to “plant the seed” for co-production (Interview 2, UK-based). Another participant mentioned that although the NMHP had already specified their needs for the proposal, “there was a lot of openness to adding new ideas, adding new sub studies, and, and all of this really” (Interview 11, Lebanon-based). However, one participant mentioned that “the research got quite heavily redesigned” during the kick-off meeting due to the fact that co-production was integrated after the proposal stage:

“It was like we had to, kind of, start the whole the process from scratch, and I think if those discussions could have happened before…I know time deadlines and one thing it’s not always possible, but I think one take away is really trying to start the co-production before you put the proposal in” (Interview 3, UK-based).

#### Design

Multiple participants who had been involved in the design of protocols and tools discussed how the process felt inclusive and collaborative:

“I felt like I had a say, in like designing the protocols and everything like that. I really felt like genuinely what I had to say was taken into consideration” (Interview 1, Lebanon-based)

“[F]or instance, the topic guides… we were reading and giving opinions about every sentence, every question” (Interview 10, Lebanon-based).

“[I]t felt like it was an inclusive process. The researchers from War Child were quite involved in drafting and reviewing the questions, and helping translate them, and making sure that the terms that we were using in Arabic were really aligning to what we were trying to get at (Interview 6, UK-based).

However, some participants expressed uncertainty about the degree of consultation with other partners during the protocol development, stating that “sometimes there’s a bigger consideration for the Ministry, instead of watching to the design of the protocols” (Interview 10, Lebanon-based). Another participant had a similar impression of the study design process: “the idea I get is that it’s not that co-produced” (Interview 8, Lebanon-based).

Participants gave examples about how the design of the research was also flexible, allowing partners to incorporate aspects they were particularly interested in. For example, the NMHP wanted different qualitative methods to be used so the financing workstream incorporated narrative story-telling as a method. Similarly, War Child were interested in exploring issues of informed consent, so this was built into a revision of the participation work. When the Beirut Blast occurred in August 2020, the participation work was also revised to include an additional component focused on this aspect, in response to the interests of the Lebanon partners.

Participants also reflected on the challenges they faced during the design process. One participant observed that it felt like the protocol development process was “too deliberative and too inclusive.” They suggested it would have been more efficient to have less people involved in developing the draft and having others give comments, rather than having so many involved from the outset. They also raised that decision-making felt slow and “it wasn’t clear who ultimately had the authority to just say that we’re doing this” (Interview 3, UK-based). This perspective raises questions about how decision-making occurs within co-produced research—specifically which decisions require everyone, how to ensure decisions are timely, and also how to bring people on board to this different way of working.

One participant also reflected on the time required to co-produce: “[T]o collaborate and to be inclusive takes time and requires being flexible and sometimes the deadlines had to shift a bit to allow for everyone to have a chance to review and participate” (Interview 6, UK-based). This participant also reflected that the protocol and tool development process could have benefited from more engagement and responsiveness from senior people who occupied higher-level positions in partner organizations: “I think that some of the senior people who had to review and sign it off were less engaged and not as responsive as they could be” (Interview 6, UK-based). While some of the lead actors from each partner organisation were involved in protocol and tool development, others were not; however, this aspect could have been strengthened to fully capture all partner perspectives. Additionally, one participant suggested that some staff (specifically those who conducted interviews) could have been involved in drafting topic guides from the outset—and not only reviewing them—to be more inclusive (Interview 1, Lebanon-based).

#### Data Collection

During interviews, participants discussed the way the teams pivoted to conducting remote data collection due to COVID-19. The flexibility within the GOAL project to stagger timeframes for data collection was mentioned as a positive compared to other projects where a lot of data often has to be collected in a short period and results in casual staff being hired to meet deadlines (Interview 11, Lebanon-based). Participants discussed the importance of team members who collected data “being credited for the knowledge production” they were involved in (Interview 1, Lebanon-based).

#### Analysis and Writing

At the time of interviews, only the governance work package had been engaged in analysis and writing, so the reflections in this section are limited to those team members. Multiple participants mentioned collaborative coding as a stand-out approach to co-production, in which the team members who had collected data were also involved in analysing and coding transcripts using the Dedoose software. The process of collaborative coding is described in detail elsewhere (Zreik et al., 2022). This inclusion of multiple actors in coding—specifically team members from the Lebanon-based NGO alongside the UK academic partner—was described as a means of “giving credit where credit is due” (Interview 1, Lebanon-based). Collaborative coding allowed team members who had not done analysis previously to be involved in that process, improved future interview skills, and increased understanding of the “full process” of research (Interview 11, Lebanon-based). One participant discussed how this approach to coding allowed them to “have the same power to give our input or to give our opinion or to discuss and to talk about our ideas about the transcript.” This participant added that this meant that “[n]o matter what is your experiences, what is your status, what is your position, let’s say, in this team or in this organisation, you have the same power to have an input, to have something to say” (Interview 14, Lebanon-based).

During the analysis and writing process, participants also described changing their normal ways of obtaining feedback to make it easier for all co-authors to collaborate and give feedback. One participant reflected:

“[W]e realised that just sending [documents] along for comments, maybe it wasn’t the most accessible way to get feedback. So we had a little meeting, where we went over the main points, and then we gathered like a lot of feedback… challenging power dynamics, but not just challenging them, like intellectually, but like actually making accommodations and making changes to make sure everyone can actually participate” (Interview 1, Lebanon-based).

Authorship was also discussed by multiple participants. In the GOAL project, multiple partners have been given the opportunity to lead academic outputs. There were a few mentions of confusion about how authorship decisions were made and communicated in interviews and the feedback session, however, as noted by participants, these decisions were outlined in Sharepoint for the GOAL team members to access. Participants also discussed ongoing plans to communicate findings to research participants.

### Future Opportunities for Co-Production

Participants also discussed future opportunities for improving how co-production is integrated. Multiple participants discussed the importance of enabling a broader group to influence the design of research questions. Participants also discussed how clarifying decision-making hierarchies might make it clearer about who is able to take decisions. Participants discussed the importance of having additional opportunities for reflection on co-production and more interaction in general between GOAL stakeholders throughout the process (beyond the Management and Advisory Group meetings). There was also discussion of how to sustain the partnership after GOAL.

## Discussion

Our findings draw attention to the complexities of co-producing research, particularly in humanitarian settings. Despite conducting training, developing a co-production plan, and integrating co-production principles into different workstreams, partners had mixed understandings of what co-production entails. Further, efforts to promote research co-production occurred among a small group of the research partners rather than consistently across all partners. Individual actors lower in their organizational hierarchies tended to be more involved in co-production, while co-production on the level of governance, financing, and partnerships between organizations was mostly discussed by more senior individuals. This aligns with Oliver and colleagues’ (2019) experience that co-production tends to involve people at similar levels engaging with each other to co-produce rather than spanning all levels of a hierarchy. This finding also suggests that it is not just power hierarchies between different partners that are relevant to consider when co-producing research, but also power *within* organisations.

We find some of Davies and colleagues’ (2020) reflection questions helpful in thinking through the implications of not all partners being on the same page about what co-production might mean for GOAL. They suggest the importance of asking: “Am I (is everyone) clear about what co-production means (in the context of our specific project) and why we think it is useful for our project?” They also suggest asking: “When is it most important to use co-production in our project, as a whole or segments of the research process?” (3–4)

Drawing on these reflection questions, the experience from the GOAL project is that more work could be done at the outset of co-produced projects to create shared expectations for participation. This also ensures that team members who are not ordinarily invited to share their opinions can be better-prepared as new opportunities are created for their decision-making. Taking this initial step to discuss expectations ensures research team members don’t feel put on the spot to contribute, and also that more thinking can be done about the best mode/approach for such contributions - instead of assuming that everyone will feel comfortable contributing in the same, ordinary ways (such as providing feedback into a document). As Davies et al. (2020) suggest, reflecting on which components require co-production is important—and may facilitate a more meaningful, intentional process instead of less concrete aspirations to co-produce everything.

Creating more opportunities for the larger research team to reflect on power is also an important lesson from our experience in GOAL, as part of ensuring co-production efforts are not confined to a small group but deeply embedded throughout the research collaboration. We draw on the work of Lokot and Wake (2021b) in emphasizing the importance of considering power dynamics within research collaborations. However, this study suggests it is also important to recognize the practical and contextual limitations—such as limited electricity access, economic and political events and time—to having co-production more embedded across a research partnership. In our study, participants recognized that having some components of the research more co-produced than others may be a function of who has time to contribute to leading particular aspects. Such practices may not necessarily be about power or lack of equity, but also reflects the availability of stakeholders to co-produce. Expecting humanitarian actors whose normal work is focused on implementation, to engage in the “slow,” reflexive work of co-production (Miles et al., 2018) may not always be realistic, especially if the expectation is for people to participate in proposal development without funding yet being received. As such, it is important that transparent conversations occur to decide together about who is practically able to be involved at each stage; this may mean engagement is not always equal but that the co-production process is flexible to take into account practical realities.

Although the proposal development process was inclusive, many felt co-production could have been brought in earlier, instead of only from the GOAL kick-off meeting. Incorporating co-production from the outset may be a good way of countering the idea that it has been included tokenistically (Lokot & Wake, 2021a). For co-production to be meaningful, significant investment through funding, resourcing, and capacity is needed, however may be limited by institutional or donor structures (Flinders et al., 2016; Kimbell & Julier, 2019), emphasising the importance of access to seed funding to invest in co-producing proposal development. Similarly, participation of service users, especially refugees may be limited by structural constraints such as labor laws, limiting the extent of co-production with the community. However, our study identified that collaborative coding and analysis was a key success within GOAL. Collaborative coding and analysis may be a helpful first step for researchers wanting to integrate co-production principles (Zreik et al., 2022).

Our study is limited by the fact that as co-authors, we were also part of the GOAL research programme, which may have affected how interview participants and other GOAL team members interacted with us, including their willingness to discuss challenges within GOAL. We tried to mitigate against this by using Jamboard to facilitate anonymous participation, but it is possible that people still felt uncomfortable to respond honestly. We were also limited by the fact that some activities in GOAL were yet to be completed, including analysis and write-up. This meant that most reflections were focused on the scoping/design and data collection phases of research.

## Conclusion

In this reflection paper, we discuss the role of co-production within a multi-partner research collaboration in Lebanon. We contribute important insights on the everyday, micro-level interactions as well as broader power dynamics involved in operationalizing co-production principles in a crisis-affected setting. Our study indicates the importance of ensuring all stakeholders within a co-production effort understand the scope of co-production, and that efforts to co-produce should not be siloed, or relegated to the responsibility of more junior staff. We find that establishing shared expectations for participation could help ensure stakeholders have the same understanding of what co-production means. However, our study highlights that even with a broad commitment to co-production, in practice it is challenging for all stakeholders to implement a co-production approach. The reality is that some stakeholders have more time for co-production than others, thus co-production may not always mean equal contributions from each stakeholder. For a humanitarian setting like Lebanon, we also find that contextual factors such as electricity access and political and economic upheaval will inevitably impact co-production, suggesting co-production may be even more challenging in such conditions. We recognize co-production requires significant time and financial commitment, however, if such resources are limited, deciding only to integrate co-production principles at specific times—at the beginning of a research collaboration and during coding/analysis, for example—may be more pragmatic. Future projects guided by co-production principles could allow a wider range of stakeholders to be involved in shaping the research questions, could clarify decision-making roles and hierarchies, and could include more opportunities for reflection on power dynamics within research collaborations.
